# Tuberculosis and Risk of Emphysema among US Adults in the NHANES I Epidemiologic Follow-Up Study Cohort, 1971–1992

**DOI:** 10.3390/epidemiologia4040044

**Published:** 2023-12-05

**Authors:** Anita Joshi, L. Joseph Su, Mohammed S. Orloff

**Affiliations:** 1 Department of Epidemiology, Fay W. Boozman College of Public Health, University of Arkansas for Medical Sciences, Little Rock, AR 72205, USA; 2 Winthrop P. Rockefeller Cancer Institute, University of Arkansas for Medical Sciences, Little Rock, AR 72205, USA; 3 Center for the Study of Tobacco, Department Health Behavior and Health Education, Fay W. Boozman College of Public Health, University of Arkansas for Medical Sciences, Little Rock, AR 72205, USA

**Keywords:** Tuberculosis (TB), emphysema, latent tuberculosis infection (LTBI), NHANES

## Abstract

(1) Background: History of TB is a known risk factor for long-term respiratory impairment affecting lung functions in both restrictive and obstructive lung disease. (2) Methods: We analyzed data from the NHANES I Epidemiologic Follow-up Study (NHEFS), a longitudinal study conducted on a noninstitutionalized adult US population aged 25–74 years. Approximately 93 percent of the original NHANES I cohort was successfully traced by the end of the survey period and was available for analysis. The final adjusted model included age groups, gender, family income, lifetime smoking, body mass index (BMI), and frequency of alcohol consumption as potential confounders. (3) Results: The estimated hazards ratio of developing emphysema during follow-up for individuals with a past diagnosis of TB was 54% lower (95% CI = 0.35, 0.61) that that in individuals with no past TB, after controlling for potential confounders and using proportional hazards regression appropriate to the complex sample design. The association, however, was not statistically significant (HR = 0.86, *p*-value = 0.38) when only a self-reported history of TB was considered as the exposure in an unadjusted model. (4) Conclusions: Tuberculosis (self-reported or LTBI) was strongly (but inversely) associated with emphysema incidence. The association was not statistically significant with only a self-reported history of TB as exposure.

## 1. Introduction

Pulmonary emphysema is a pathological term that describes abnormal and permanent dilatation of the airspaces distal to the terminal bronchioles and is often accompanied by destruction of the airspace walls and airflow limitation [1]. It is a subtype of chronic obstructive pulmonary disease (COPD), along with chronic bronchitis, and can lead to persistent respiratory symptoms [1]. Tobacco smoking is a major known cause of both emphysema and COPD. Genetic factors, such as α1-antitrypsin protein deficiency and telomere length, have also been implicated [2], although a 10-year case–control study found no association between emphysematous changes in the lungs and telomere length [3]. The group of older (>60 years of age), asymptomatic individuals with subclinical pulmonary emphysema were generally male and smokers with low body mass index, suggesting that age, sex, and BMI are important risk factors for subclinical emphysema [3].

Tuberculosis (TB) is an infectious disease caused by the *Mycobacterium tuberculosis* complex and can cause disease in almost any area of the body, but primarily affects the lungs. Although a history of TB is a known risk factor for long-term respiratory impairment affecting lung functions in both restrictive and obstructive lung disease [4], there is minimal research associated specifically with emphysema. The link between TB and the risk of developing emphysema is unclear.

Reports have shown that the long-term effects of TB infection on the lungs can lead to chronic inflammation with anatomical changes that mimic emphysema/COPD [5,6]. Along these lines, data from the annual Korean National Health and Nutrition Examination Surveys for adults aged 40 years and older were analyzed by Jung and colleagues. They found that a past history of physician-diagnosed prior pulmonary TB (mean of 29.0 years) was independently associated with impaired pulmonary lung function after adjusting for other risk factors such as age, gender, asthma diagnosis, and smoking [7]. Similarly, Willcox and colleagues found evidence of airway obstruction in 48 (68.0%) of 71 subjects that who been successfully treated for TB up to 16 years prior to being assessed for pulmonary functions [8]. Another retrospective review of 1784 COPD patients by Park et al. found that tuberculosis infection negatively affects the severity of obstructive lung disease [9]. Thus, most prior studies have assessed the association between symptomatic TB and emphysema or COPD.

Not everyone infected with *Mycobacterium tuberculosis* develops TB disease. Individuals with noninfectious latent TB (LTBI) are asymptomatic and are identified only with a positive reaction to the tuberculin skin test (TST) or interferon-γ release assay (IGRA) tests. The prevalence of LTBI as determined using TST measurements collected as part of the National Health and Nutrition Examination Survey (NHANES, 1999–2000) was estimated to be 4.2% in the noninstitutionalized civilian population in the United States [10]. In 2019, there were 8,916 reported TB cases in the United States alone, with an estimated 13 million people living with LTBI, and COPD remained the fourth most common cause of death [11]. With the knowledge that the lifetime risk for LTBI individuals developing TB disease is known to be 5 to 10 percent [12], any association between a history of TB (clinical or latent) and emphysema has clinical implications.

The objective of this analysis is to assess if a history of tuberculosis (self-reported, or a diagnosis of LTBI at the time of initial interview and examination) is associated with an increased risk of being diagnosed with emphysema in the follow-up period while adjusting for commonly known confounders. We also assessed if there was an association between the subgroup of self-reported TB (excluding LTBI) and the development of emphysema. To the best of our knowledge, this is the first large retrospective cohort study using a well-established noninstitutionalized cohort of US adults to study the association between a history of clinical TB and/or the presence of LTBI with the development of emphysema during a 20-year follow-up period.

## 2. Materials and Methods

### 2.1. Study Population

The first National Health and Nutrition Examination Survey (NHANES I) Epidemiologic Follow-up Study (NHEFS) is a longitudinal study conducted on a baseline adult population aged 25 to 74 years who were examined in the NHANES I survey. Approximately 93 percent of the cohort was successfully traced by the end of the survey period. Comprising of four follow-up surveys, the first wave of data collection, the 1982–1984 NHEFS, included all adults between 25 and 74 years of age at their NHANES I examination (*n* = 14,407). The second wave of data collection in the 1986 NHEFS included members of the NHEFS cohort who were 55–74 years of age at their baseline examination and not known to be deceased at the 1982–1984 NHEFS (*n* = 3980). The 1987 NHEFS was conducted for the entire non-deceased NHEFS cohort (*n* = 11,750). The final 1992 follow-up included the entire non-deceased cohort (*n* = 11,195).

The NHANES I (1971–1974) collected data from a national probability sample of the noninstitutionalized civilian United States population between the ages of 1 and 74 and included standardized questionnaires and medical examinations covering various health-related topics. Although the original sample was augmented in 1974–1975, baseline medical examination and related information were available for only 14,407 (70 percent) subjects, and this was the cohort that was used in this study.

### 2.2. Identification of Emphysema Cases at Baseline for Exclusion

The NHANES I survey, and medical examination recorded chest X-ray, spirometry, and self-reported emphysema, where the subject reported having been told by their doctor that they had chronic bronchitis or emphysema. Individuals with the following criteria were identified as prevalent cases of emphysema and excluded from analysis at the beginning of the follow-up period. This resulted in a final sample size of 13,340.

- An explicit presumptive diagnosis of emphysema on chest X-ray readings;
- Possible evidence of emphysema with chest X-ray findings combined with spirometry results not suggestive of restrictive lung disease;
- An obstructive pattern on spirometry with self-reported chronic bronchitis/emphysema;
- All self-reported cases who reported that they still have the condition, when the report could be corroborated with a chest X-ray.

A restrictive pattern on spirometry ruled out a diagnosis of emphysema. A detailed workflow diagram explaining this criterion is available in Supplemental Figure S1. Decision mapping also shown in Appendix A

### 2.3. Identification of Past or Current Tuberculosis at Baseline

The NHANES I survey asked survey respondents about past tuberculosis diagnosis. Tuberculin tests were administered only to a subset of the sample (*n* = 6913). Participants with a history of a positive reaction, or isoniazid (INH) prophylaxis were not administered the tuberculin skin tests; it was assumed that persons in this group would have had a positive reaction if the test had been administered. Simultaneous skin testing with the PPD-S tuberculin antigen and PPD-B antigen permitted differentiation between reactivity caused by infection with M. tuberculosis and other mycobacteria [13]. A positive tuberculin test is a reaction to PPD-S equal to or greater than 10 mm in diameter or a reaction to PPD-S of 5–9 mm with a reading of PPD-S at least 2 mm greater than the PPD-B reading [13]. Only 3.7% subjects in the baseline cohort were thus identified as having past or current TB. All individuals not meeting the criteria for a positive tuberculin test and not reporting past TB infection were considered to be negative for past and current TB and LTBI.

### 2.4. Other Covariates Measured at Baseline

Of the characteristics measured at the NHANES I interview and examination, age, gender, race, family income, education level, the number of individuals per room per household (proxy for overcrowding), and urban versus rural (proxy to air pollution) were some of the demographic variables analyzed as covariates. We also analyzed current/lifetime smoking and the frequency of alcohol consumption. Several risk factors, such as a history of respiratory conditions during childhood, vitamin D intake, and occupational exposure to mineral and cotton dust, have been associated with obstructive lung disease, TB, or both. These were not assessed in this study because this information was unavailable in the NHANES data. Although not directly associated with emphysema, some studies have found substantial correlations between airway obstruction and physical activity whereas other studies have been unable to establish the same relationship [14,15].

### 2.5. Identification of Incident Cases

Incident cases of emphysema were ascertained using responses from interviews, health care facility medical records for those that reported having had an overnight stay in a health care facility after their initial interview or examination, and death certificates. The follow-up cohort excluded individuals with an emphysema diagnosis at the time of their initial survey or medical examination.

For the 1982–1984 follow-up interview, interviews with the participant or a proxy were completed for 12,220 (85% of the original cohort) subjects. If the subject self-reported being told they had emphysema, they were asked for the year they were first told they had the condition and if, since 1970, they had stayed overnight in a hospital for the condition (emphysema in this case). The question was not asked in the 1986, 1987, or 1992 follow-up interviews. Emphysema cases for these years were determined using information available in facility files or death certificates. The incidence in our study population was similar to other estimates [16,17].

To extract cases of Emphysema from the health care facility files, we conducted a character string search for “Emphysema” on all condition codes. In the death records, incidence cases were individuals identified with emphysema as an underlying cause of death (ICD-9 codes of 492, 496). Any individual with a date of diagnosis after the first initial interview and examination was included as an incidence case.

### 2.6. Analytical Methods

Data were analyzed using SAS 9.4 (SAS institute Inc., Cary, NC, USA) software for all analyses. The Proc survey logistic method was used to calculate populate estimates. Sample weights, stratification, and clustering, given the complex survey design of NHANES I, were used to calculate population estimates and their standard errors [18]. All two-tailed *p*-values for the bivariate analysis were based on population estimates; a value of 0.05 or less was considered significant. For continuous variables, after testing for normality, the means in the groups were compared using the *t* tests. To generate *p*-values for categorical variables, we used either the chi-square or the CMH (Cochran–Mantel–Haenszel) depending on the number of levels of the independent variable.

Past or current TB was determined at baseline. For incidence cases of emphysema, the length of follow-up was calculated in months between the diagnosis date and the date of initial examination. Censoring time was the date of death when the subject died due to an underlying cause other than emphysema or the last date of follow-up when lost to follow-up. Individuals whose follow-up period ended before 1 January 1992, were coded as censored. The final vital status as of the 1992 interview is shown in Table 1 [19].

The “Other” race category had only 5 cases of emphysema and was combined with the “African American” race category. For each predictor variable, the group with the lowest risk category was used as the reference group. The covariates considered for inclusion during model building were race (reference = “White”), age groups in 10-year increments (reference = “25–34 years”), education level (reference = “One year college or more”), lifetime smoking of greater than 100 cigarettes, BMI category (reference = “Normal”), gender activity (reference = “Male”), family income (reference = “$25,000 and over”), and region (reference = “Northeast”). Confounding at the stage of analysis was controlled using a multivariable Cox proportional hazards regression model using the SAS SURVEYPHREG procedure that incorporated the complex survey design and sampling weights. Backward and stepwise selection methods were employed to determine which variable to include in the final model. Terms were included in the final model if they were statistically significant at *p* < 0.05 or if the goodness of fit statistics decreased by a significant amount. The variables in the final model were explored for possible confounding and effect modification.

The adjusted model included age groups, gender, family income, lifetime smoking, BMI, and alcohol consumption frequency categorized as heavy drinking versus moderate/rare drinking and a reference group of individuals who reported never drinking. Interaction was explored using appropriate interaction terms within the proportional hazards model. The interaction term was retained in the final model only if it was significant with a *p*-value of <0.05.

## 3. Results

The final sample size after excluding 1067 prevalent cases of emphysema was 13,340. Using the criteria described in detail in the Methods section, 994 new cases of emphysema were identified during the 20-year follow-up period ending in 1992. The number of new cases by year of follow-up is shown in Table 2. The mean length of follow-up was 221.8 months with a standard error of ±0.3 months. 

The characteristics in Table 3 reflect the population-based estimated percentages for categorical variables incorporating survey design specifications (adjusted weights, stratification, multistage cluster sampling), including oversampling of a certain population in the NHANES cohort. The group that developed emphysema during follow-up included significantly higher proportion of whites (94.3% versus 88.5%) and a significantly lower proportion of African Americans (5.4% versus 10.2%). They were also slightly older, reported more lifetime smoking (77.6% versus 59.8%), and were less educated with less reported family income than the group that did not develop emphysema. The groups were not significantly different by gender, urban versus rural, region, or type of living quarters (housing units or other). The groups were similar in the proportion of individuals diagnosed with past or current TB (3.6% versus 2.9%, *p*-value = 0.418). The number of people per room per household was used as a proxy for overcrowding and individuals living in a house with a range or cookstove as a proxy for indoor air pollutant exposure. However, one-fifth of the responses in both these categories were missing. The majority (>99.0%) of the individuals who did respond said yes to having a range/cooking stove at home, thus reducing the likelihood of pollution from that source. Similarly, the median number of people per household was three; hence, we concluded that household crowding was not a major factor in our analysis. These variables during the bivariate analysis were, therefore, not included in the multivariable analysis.

The unadjusted model exploring the association between TB and new diagnosis of emphysema was significant (Hazard ratio = 0.51; *p* value <0.0001). The association was significant even after adjusting for all relevant factors (hazard ratio = 0.46). The association, however, was no longer statistically significant (HR = 0.86, *p*-value = 0.38) when only the self-reported history of TB was considered as the exposure in an unadjusted model.

Based on multivariable proportional hazards regression for a complex survey design, the population-estimated hazard ratio of developing emphysema during follow-up in individuals who had a past diagnosis of TB was 54% lower (95% CI = 0.35, 0.61) than that in individuals with no past TB, after controlling for gender, age, family income, lifetime smoking (>100 cigarettes), alcohol consumption, and BMI (Table 4). Females had a 19% greater likelihood of developing emphysema than males, and individuals who reported smoking at least 100 or more cigarettes in their lifetime were 12% more likely to develop emphysema than individuals who smoked less than 100 cigarettes. Having a family income of less than $25,000 was associated with a lower hazard of developing emphysema, even in families in the lowest income category. Alcohol consumption was not a significant predictor of emphysema risk. However, adding it to the model reduced the goodness of fit statistics by a significant amount, so the variable was included in the final model. Heavy drinking was defined as drinking every day, just about every day, or 2–3 times a week. Heavy drinking is known to reduce the levels of glutathione, an antioxidant that protects the lungs from smoke damage. In addition, regular drinking can damage the mucociliary transport system, which impedes clearing of mucus and other contaminants [20].

Low BMI (<18.5 lb./inch^2^) was associated with a reduced hazard of being diagnosed with emphysema in the future by 56% (HR = 0.44, 95% CI = 0.38, 0.51). High BMI (>25 lb./inch^2^), on the other hand, increased the risk slightly by 11% (HR = 1.11, 95% CI = 1.02, 1.21). Subjects in the age group 45–54 years were slightly protected with a hazard ratio of 0.89 (95% CI = 0.82, 0.97), and subjects in the age group 65 and older at baseline were at slightly higher risk when compared with the youngest age group of 25–34 years. This risk distribution makes sense because the risk of emphysema due to a TB diagnosis will remain high in younger age groups that may be diagnosed with TB disease at a younger age. None of the other age groups displayed a statistically significant difference in the risk of emphysema.

**Figure 1:**
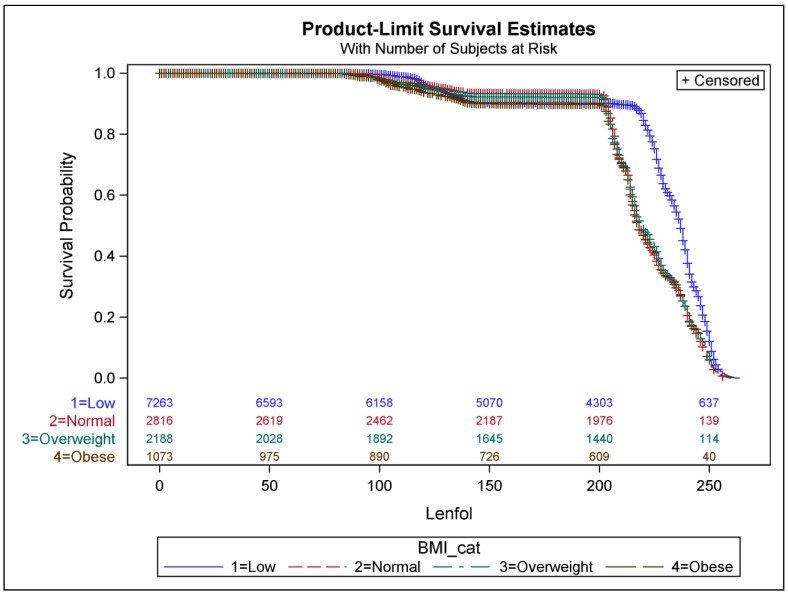
Survival probability (risk of developing emphysema) by BMI category.

We observed a significant interaction between BMI category and past TB. Figure 1 depicts the survival probability of the event (developing emphysema) by BMI category. As seen in Figure 1, individuals with a lower BMI developed emphysema significantly later than those who were normal, overweight, or obese. Because weight loss can sometimes improve breathing, this is one way that the clinical diagnosis may be delayed. However, further analysis of this effect heterogeneity is necessary to understand the biological mechanism behind the effect.

## 4. Discussion

Tuberculosis (self-reported or LTBI) was strongly (but inversely) associated with emphysema incidence in a noninstitutionalized adult US population after adjusting for factors including lifetime smoking exposure, age, gender, family income, alcohol consumption, and BMI. The direction of association seems to contradict our prior knowledge of the biological basis of developing obstructive/restrictive lung disease after developing TB. Several population-based studies have demonstrated that a history of TB increases the risk of airflow obstruction and COPD [6,21]. Our findings may partially reflect the heterogeneity in lung damage seen after TB, which is attributed to the host immune response to the infection [22]. Pulmonary function testing in individuals with TB demonstrates variable patterns, as well as severity of impairment [22].

Another possible explanation for this finding could be that a large majority (66.1%) of the 492 TB cases at baseline were latent TB infections (LTBI) diagnosed based on tuberculin testing rather than clinical symptoms or chest X-ray findings. Survey respondents who self-reported being told they have or had TB did not have a higher risk of developing emphysema when compared with those who reported never being told they have or had TB (HR = 0.86, 95% CI = (0.61 to 1.21). The estimate, albeit not significant and with a wider confidence interval, is therefore consistent in directionality with our original findings. We conducted a further sensitivity analysis using LTBI as the sole exposure group, and the results remained consistent (see Table 5).

Pasipanodya et al. conducted a prospective case–control study comparing pulmonary function tests for 107 patients with pulmonary tuberculosis disease who had completed at least 20 weeks of therapy and a control group of 210 individuals with LTBI [23]. Interestingly, after adjusting for several risk factors, they found that the group with TB was 5.4 times more likely to have abnormal pulmonary function test results than the LTBI group (*p* > 0.001; 95% CI = 2.98–9.68). Interestingly, their findings indicated that older subjects born in the United States were more likely to have lung function impairment than younger or foreign-born subjects [23]. It is possible that the very diagnosis of LTBI may promote behavioral modifications to decrease exposure to noxious stimuli that might further decrease lung functionality. There is also the possibility of residual confounding from smoking. Although lifetime smoking was adjusted for in the final model, current smoking and smoking exposure in the follow-up period just prior to the diagnosis of emphysema were not assessed.

Subjects who reported having smoked at least 100 cigarettes or more in their lifetime were at a higher risk of developing emphysema at follow-up after controlling for the other factors. Information on family history of lung diseases and exposure to secondhand smoking was not available in NHANES I. Given that secondhand smoke exposure and its effects on lung function are poorly defined and understood, we decided to not use secondhand smoke in our analysis.

We were also limited by being unable to study the presence of α1-antitrypsin protein deficiency, which is now known to be implicated with an earlier onset of COPD, disproportionate to smoking history and other genetic factors, such as telomere length [2]. Telomeres are nucleoprotein structures at the end of each chromosome that protect the chromosomal ends from degradation. Although the length is genetically determined, there is progressive shortening with cell division, and short telomeres are considered a marker of aging in cells. A 2011 study in mice indicated that short telomeres lower the threshold of smoking-induced lung damage, thus raising the potential for telomere length as a genetic susceptibility factor in age-related onset of emphysema [24].

TB cases beyond baseline interview and examination were not assessed. We assumed that even if there were a few individuals diagnosed with TB during follow-up, it likely would not affect their risk of being diagnosed with emphysema during the follow-up period ending in 1992. It is a slow and variable process from the initial pathophysiological response due to TB infection leading to the destruction of the lung tissue, eventually resulting in emphysema.

Another limitation is the incomplete ascertainment of outcome because the follow-up questionnaire did not ask a specific question about self-reported emphysema or TB after the 1986–1987 follow-up. The events of emphysema diagnosis after the 1986–1987 survey were ascertained using medical records from an overnight stay at a health care facility. Therefore, we were only able to capture cases of emphysema that were severe enough to require at least an overnight stay. Very few incidence cases of emphysema are diagnosed through a visit to a health care facility, and attempting to identify new cases solely via health care facility data is an underestimate of the actual incidence. As mentioned in a previous section, data for 7180 COPD patients obtained from the 2012 Behavioral Risk Factor Surveillance System (BRFSS) survey revealed that among diagnosed COPD patients, only 16.5% had ED visits or hospitalization in the previous year [16]. However, the incidence in our population (7.4%) is close to, or even slightly higher than, the 2018 US CDC-estimated combined prevalence of chronic bronchitis and emphysema (5.2%) based on self-reporting [17].

This study has several strengths. To our knowledge, this is one of the first population-based follow-up large case cohort study of its kind to assess this association. The NHANES I data provide a wealth of information on the prevalence of health conditions and risk factors, and the NHEFS data are extremely useful when investigating the association between factors measured at baseline and the development of specific health conditions during the 20-year follow-up. The NHANES I and NHEFS have been designed and documented extensively, with every step taken to reduce loss to follow-up, thus minimizing bias. Spirometry, chest X-ray, tuberculin testing, and other factors based on body measurements and laboratory data were all conducted with standardized and validated methods to the extent possible.

Females were 1.19 times more likely to develop emphysema during follow-up. According to the CDC, there are several reasons why COPD might affect women differently than men. Delayed diagnosis in females and their greater vulnerability to the effects of tobacco and other harmful substances, such as indoor air pollution, are just a few possible explanations for this effect. Women who smoke tend to develop COPD at younger ages and with lower levels of smoking than men who smoke [25]. Delayed diagnoses associated with more advanced disease at diagnosis and less effective treatment strategies may also explain the reason for differential response to treatment and higher mortality in females [25].

Significant interaction by BMI category requires further research to explore and explain the role of BMI as an effect modifier for the association between TB and emphysema. Undernutrition is a known risk factor for the development of TB, and conversely, active TB itself causes wasting [26]. Similarly, individuals who develop emphysema are prone to weight loss, leading to low BMI (<18.5 kg/m^2^) [27]. Being overweight and obese, however, is not a predisposing factor for the development of emphysema [27].

This study has implications beyond emphysema incidence in the US population. The burden of TB disease is extremely high in countries outside US. The bacteria have survived for over tens of thousands of years, [28] and in 2017, they were estimated to have infected nearly 2 billion people worldwide. More than 10 million new cases of TB are diagnosed each year, and a large proportion of the world’s population are latent carriers with a risk of developing active disease in the future [28]. With ever-increasing air pollution and increasing respiratory illnesses in childhood, any association between history of TB and emphysema has clinical implications. In countries and communities where TB, pollution, and undernutrition are prevalent, the cases of obstructive lung diseases attributable to TB can be substantial and need to be urgently addressed.

## Figures and Tables

**Table 1:** Current vital status of subjects in 1992 by year of follow-up.

Vital Status (1992)	(Year of Follow-Up and “Last Known Alive”)					
	1982–1984	1986	1987	1992	Inapplicable *	Total
Alive	47	5	356	7926	0	8334
Dead	1758	557	496	1244	0	4055
Unknown	0	0	0	0	519	519
Traced—directly	5	1	2	237	0	245
Traced—indirectly	0	0	124	63	0	187
Total	1810	563	978	9470	519	13,340

* Subject’s vital status unknown in all eligible follow-up surveys.

**Table 2:** New emphysema diagnosis by year of last follow-up. (Includes self-reported and medical/death records indicating diagnosis).

Year of Diagnosis ^¥^	Incident Cases of Emphysema
1973–1975	4
1977–1981	8
1982	160
1983	775
1984	20
1985–1987	13
1988–1992	14
Total incident cases	994

^¥^ Years have been combined for reporting purposes only.

**Table 3:** Characteristics (population-estimated percentages ^a^) of participants who developed emphysema during follow-up (*n* = 994), 1971–1992 and those who did not (*n* = 12,346).

Characteristics	Individuals Who Developed Emphysema (*n* = 994)		Individuals Who Did Not Develop Emphysema (*n* =12,346)		*p* Value
	%	95% CI	%	95% CI	
**Race**					<0.001
Whites	94.3%	92.4, 96.2	88.5%	86.8, 90.1	
African American	5.4%	3.5, 7.3	10.2%	8.6, 11.8	
Other	0.3%	0.0, 0.8	1.3%	0.9, 1.7	
**Gender**					0.74
Females	52.4%	47.2, 57.6	53.3%	51.8, 54.9	
Male	47.6%	42.4, 52.8	46.7%	45.1, 48.2	
**Age group (** **age in years** **at time of enrollment)**					<0.001
25–34	17.9%	13.8, 22.0	29.1%	27.8, 30.4	
35–44	16.6%	13.5, 19.8	21.8%	20.6, 22.9	
45–54	23.7%	20.4, 27.1	21.9%	20.9, 22.9	
55–64	26.2%	22.2, 30.2	16.4%	15.4, 17.5	
≥65	15.5%	11.8, 19.2	10.8%	10.2, 11.4	
**Urban** versus **rural**					0.93
Urban	68.3%	63.6, 72.9	68.5%	64.9, 72.1	
Rural	31.7%	27.1, 36.4	31.5%	27.9, 35.1	
**Region**					0.83
Northeast	26.3%	19.4, 33.3	24.2%	21.3, 26.9	
Midwest	24.4%	19.4, 29.5	25.5%	22.2, 28.9	
South	24.0%	19.6, 28.5	23.7%	19.4, 27.9	
West	25.2%	20.1, 30.4	26.6%	22.3, 30.9	
**Type of living quarters**					0.63
Living in housing units	99.3%	98.2, 100.0	99.5%	99.2, 99.7	
Other units	0.7%	0.0, 1.8	0.5%	0.33, 0.76	
**Individuals living in house with a range or cookstove ^b^**					0.02
Yes	99.7%	99.4, 100.0	99.1%	98.8, 99.4	
**Number of people per room per household ^b^**					<0.0001
1	93.5%	91.5, 95.4	91.3%	90.2, 92.5	
2	6.0%	4.0, 8.0	8.4%	7.3, 9.5	
3	0.5%	0.0, 1.2	0.2%	0.1, 0.4	
4–5	0.1%	0.0, 0.2	0.0%	0.0, 0.0	
**Family income (in dollars) ^c,d^**					<0.0001
Less than $5000	21.2%	17.5, 24.8	17.5%	16.0, 18.9	
$5000–$10,000	33.1%	29.1, 37.1	26.6%	24.9, 28.3	
$10,000–$24,999	40.2%	35.4, 45.0	45.3%	43.3, 47.4	
$25,000 and over	3.1%	1.8, 4.4	6.9%	5.9, 7.9	
Missing	2.4%	1.1, 3.8	3.8%	3.2, 4.4	
**Educational attainment**					<0.0001
High school or less	66.5%	61.9, 70.9	54.1%	51.4, 56.8	
At least a year college	33.1%	28.6, 37.6	45.1%	42.4, 47.8	
Missing	0.4%	0.0, 0.8	0.8%	0.5, 1.0	
**Physical activity (recreational or otherwise)**					0.01
Very active	44.4%	40.0, 48.7	49.1%	47.5, 50.6	
Moderately active	46.5%	42.2, 50.8	43.7%	42.2, 45.1	
Quite inactive	9.2%	6.1, 12.3	7.2%	6.3, 8.0	
Missing	0.0%	0.0, 0.0	0.1%	0.0, 0.2	
**Have or had tuberculosis**					0.42
Yes	3.6%	1.9, 5.2	2.9%	2.2, 3.7	
**Lifetime smoking (smoked at least 100 or more) ^d^**					<0.0001
Yes	77.6%	73.7, 81.4	59.8%	58.2, 61.2	
**Frequency of alcohol consumption ^e^**					0.03
Everyday/Just about…	16.8%	13.1, 20.6	15.9%	14.5, 17.3	
About 2–3 times a week	12.1%	9.4, 14.8	14.3%	13.2, 15.3	
About 1–4 times a month	23.9%	19.7, 28.2	26.6%	25.2. 28.1	
≥3 but <12 times a year	10.3%	7.7, 12.8	9.4%	8.6, 10.1	
≤2 or 3 times a year	12.7%	9.9, 15.6	11.3%	10.4, 12.1	
Do not drink	23.1%	19.3, 27.0	22.4%	20.3, 24.5	
Missing	0.0%	0.0, 2.5	0.2%	0.1, 0.3	
**BMI ^f^**					0.03
Low (<18.5)	37.4%	29.7, 45.2	31.9%	25.2, 38.5	
Normal (18.5–<25)	25.9%	21.4, 30.4	31.6%	28.0, 35.1	
Overweight (25–<30)	23.8%	18.5, 29.2	25.0%	22.5, 27.5	
Obese (>=30)	12.8%	9.8, 15.9	11.6%	10.1, 13.2	
	**Mean**	**SE**	**Mean**	**SE**	***p* value**
**Age at initial interview, in years**	52.8	0.5	47.9	0.1	<0.0001
**People in household (number)**	3.1	1.8	3.6	1.9	<0.0001
**Years since TB diagnosis ^g^**	13.2	2.8	7.9	0.7	0.07

^a^ Data shown are population-based estimated percentages for categorical variables incorporating survey design specifications (adjusted weights, stratification, multistage cluster sampling). Of the 14,407 individuals in the entire NHEFS cohort, 1067 prevalent cases of emphysema (at first interview) were excluded, leaving 13,340 for follow-up. ^b^ A total of 20.1% of the responses in both these categories were missing. The number of people per room per household was used as a proxy for overcrowding. The median household size for the study population was 3.0 (range = 1.191–19). The number was calculated by dividing the total number of people in the household by the total number of rooms in the household. These variables were not included in the analysis. The majority of the individuals who did respond said yes to having a range/cooking stove at home, thus reducing the likelihood of pollution from that source. Similarly, the median number of people per household was three; hence, we concluded that household crowding was not a major factor in our analysis. ^c^ An income of $10,000 in 1973 dollars is equivalent to an income of $60,556.57 in 2020 (https://www.bls.gov/data/inflation_calculator.htm; accessed on 29 November, 2023). ^d^ At baseline, smoking information was obtained only for subjects in the detailed examination; follow-up questions from the 1982 interview were used to reconstruct smoking status at baseline for subjects who were not in the detailed sample. ^e^ Frequency of alcohol consumption—Three different categories for alcohol consumption were assigned based on responses to the question “How often do you drink?”. The question was asked to all individuals who said “Yes” to having at least one drink of beer, wine, or liquor in the past year. ^f^ Weight (lb.)/height (inch)^2^. ^g^ Median years since diagnosis of TB was 0 years with a range of 0 to 68. Many individuals were diagnosed with TB (positive PPD) at the time of initial examination.

**Table 4:** Population-estimated hazard ratios for the relation of incidence of emphysema with past tuberculosis and other risk factors among US adults, NHANES I epidemiologic follow-up study, 1971–1992 ^a,b^.

Characteristics	Adjusted Hazard Ratio	95% Confidence Interval
**Have or had tuberculosis**		
Yes	0.46	0.35, 0.61
No	1	Reference
Gender		
Female	1.19	1.11, 1.28
Male	1	Reference
**Age group (in years)**		
25–34	1	Reference
35–44	0.94	0.86, 1.02
45–54	0.89	0.82, 0.97
55–64	0.95	0.85, 1.06
≥65	1.17	1.00, 1.36
**Family income (in dollars)**		
Less than $5000	0.72	0.61, 0.83
$5000–$10,000	0.69	0.59, 0.80
$10,000–$24,999	0.81	0.71, 0.94
$25,000 and over	1	Reference
**Lifetime smoking (smoked at least 100 or more)**		
Yes	1.12	1.05, 1.20
No	1	Reference
**Frequency of alcohol consumption ^b^**		
Frequent drinkers	0.93	0.81, 1.06
Moderate/Rare drinkers	0.90	0.79, 1.02
Never drinkers	1	Reference
**BMI category ^c^**		
Low BMI (<18.5)	0.44	0.38, 0.51
Normal BMI	1	Reference
Overweight/Obese (≥25)	1.11	1.02, 1.21

^a^ Results are based on multivariable Cox proportional hazards regression analysis using the SAS SURVEYPHREG procedure. Terms were included in the final model if they were statistically significant at *p* < 0.05 or if the goodness of fit statistics decreased by a significant amount. ^b^ Alcohol consumption did not meet the criteria for inclusion in the final model if only a cut-off of *p* < 0.05 is applied. However, adding it to the model reduced the goodness of fit statistics by a significant amount. Heavy drinking was defined as drinking every day, just about every day, or 2–3 times a week. ^c^ Individuals with low BMI have decreased hazard of being diagnosed with emphysema in the future, whereas individuals who were overweight or obese were at higher risk of developing emphysema; risk was similar in the overweight and obese categories, and they combined for analysis.

**Table 5:** Findings for unadjusted analysis using different exposure groups.

Exposure Group (As Defined)	*n*	Cases of Emphysema during Follow-up	Unadjusted HR (95% CL)
Have or had TB (includes self-reported and/or LTBI, individuals with positive TST at baseline)	492	37	0.51 (0.44, 0.60)
Self-reported TB only	175	24	0.86 (0.61, 1.21) ^¥^
LTBI only	325	15	0.43 (0.26, 0.73) ^€^

^¥^ Remains nonsignificant, even after adjustment for confounders used in the original analysis. ^€^ Remains significant, even after adjustment for confounders used in the original analysis.

## Data Availability

Analyzed datasets can be made available upon approval, if required. Publicly available NHANES I and NHEFS datasets were used for the analysis and can be accessed at: NHANES I (1971–1974) (cdc.gov) and NHANES I-Epidemiologic Follow-up Study (NHEFS) (cdc.gov).

## Supplementary Materials

The following supporting information can be downloaded at: https://www.mdpi.com/2673-3986/4/4/44/s1, Supplementary Figure S1: Decision tree model depicting how prevalent cases of emphysema were determined at baseline, combining self-reporting, chest X-ray findings, and spirometry results.

## Appendix A

**Table A1:** Decision map for identification of prevalent Emphysema cases at baseline.

Rule-based decision mapping for emphysema combining information from chest X-ray findings, spirometry results, and self-reporting to determine if the individual subjects had emphysema at the time of study initiation.
If chest X-ray showed an “**Explicit presumptive diagnosis of emphysema**”, then regardless of lung function or self-reporting, the individual was flagged as having **emphysema.** - *Chest X-ray is the gold standard for emphysema diagnosis*.
2. If chest X-ray showed only a “**Possible diagnosis of emphysema**” and lung functions showed a **normal or obstructive pattern**, then the individual was flagged as having **emphysema.** - *Self-reporting is disregarded; flat diaphragm combined with either normal lung functions of obstructive pattern indicate emphysema.* 3. If chest X-ray showed only a “**Possible diagnosis of emphysema**” and lung functions showed a **mixed obstructive–restrictive pattern**, and the individual **self-reported** as having been diagnosed with emphysema, then they were flagged as having **emphysema.** - *Self-reporting considered; flat diaphragm combined with a mixed pattern on spirometry and self-reporting indicate emphysema.* 4. If chest X-ray showed only a “**Possible diagnosis of emphysema**” and lung functions showed a **restrictive pattern**, then **emphysema—ruled out.** - *Restrictive physiology makes emphysema unlikely.*
5. **Normal finding on chest X-ray** and **normal lung functions** rules out the possibility of emphysema (**emphysema—ruled out**) - *If radiographic finding does not support a diagnosis of emphysema, even in the case of self-reporting, the emphysema is not clinically relevant. Self-reporting may be in response to the symptoms of chronic bronchitis rather than emphysema.* 6. In cases where **chest X-ray findings were missing** and **lung functions were normal**, but the individual **self-reported** as having been diagnosed with emphysema, then they were flagged as having **emphysema.**
7. If **chest X-ray findings either normal or missing** and **restrictive pattern** on spirometry, then individual was flagged as having **emphysema—ruled out.** - *Restrictive physiology makes emphysema unlikely.* 8. If **chest X-ray findings either normal or missing** and **obstructive pattern** on spirometry, then individual was flagged as having **emphysema.** - *Obstructive physiology makes emphysema likely.* 9. If **chest X-ray findings either normal or missing** and **mixed obstructive–restrictive pattern** on spirometry, then self-reporting by individual was considered. The subject was flagged as having **emphysema** if they reported as having been diagnosed with emphysema. The subject was flagged as having **emphysema—ruled out** if they reported as **never** having been diagnosed with emphysema.
