# Supplementary material for: Tuberculosis and Risk of Emphysema among US Adults in the NHANES I Epidemiologic Follow-Up Study Cohort, 1971–1992
Source: Epidemiologia (Basel). 2023 Dec 5;4(4):525–37. doi: 10.3390/epidemiologia4040044 (PMC10871094; doi:10.3390/epidemiologia4040044)

Supplemental Figure S1: Decision tree model depicting how prevalent cases of emphysema were determined at baseline, combining self-reporting, chest X-ray findings, and spirometry results.

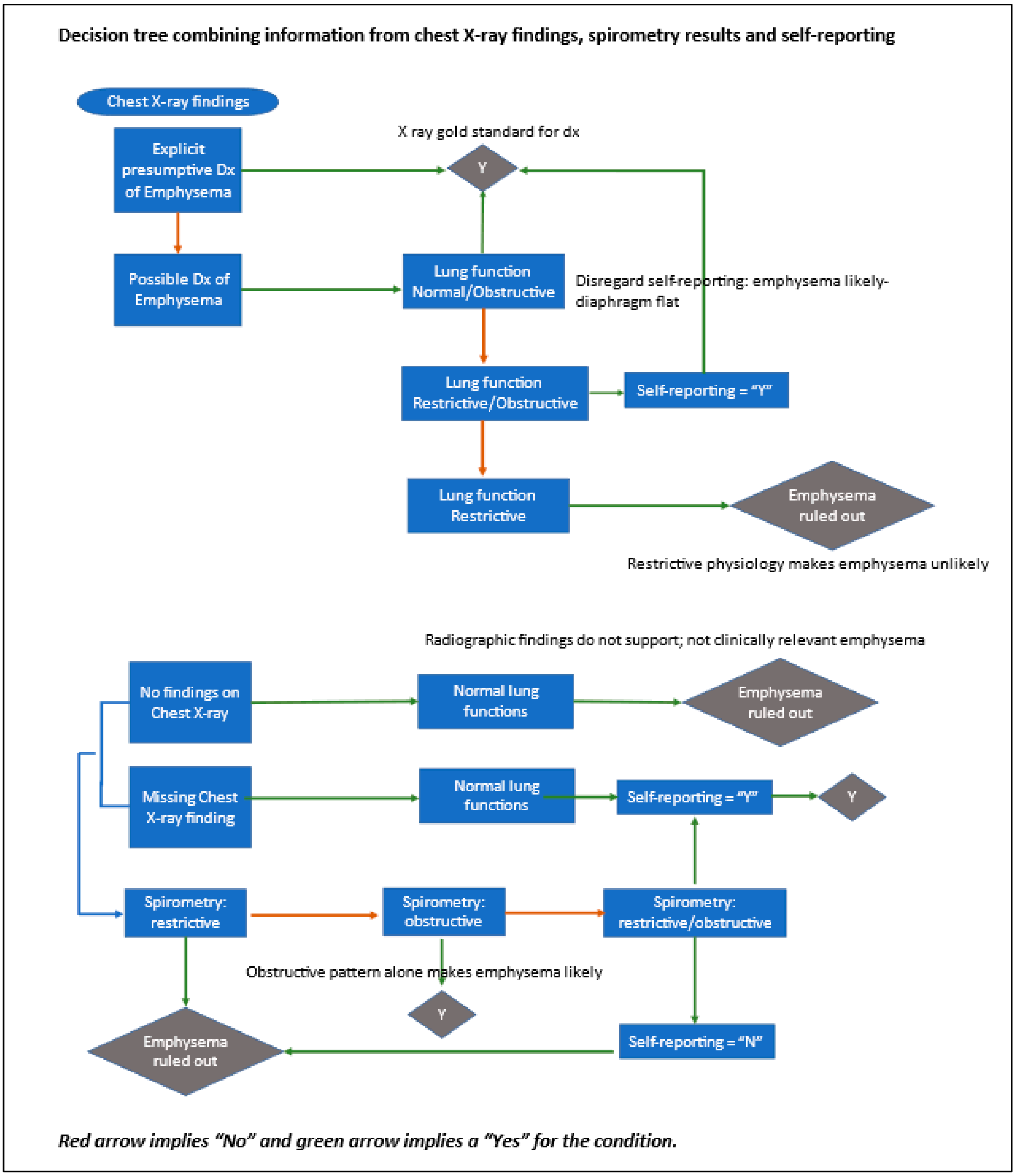

Supplement: Supplementary file 1 [file epidemiologia-04-00044-s001.zip › epidemiologia-2558214-supplementary.pdf]
